# Caste-Associated Gut Microbial Diversity and Predicted Functional Profiles in *Coptotermes formosanus*

**DOI:** 10.3390/ijms27167297

**Published:** 2026-08-15

**Authors:** Zhimeng Cao, Zhengyang Li, Hengyu Yan, Wanjiang Tang, Huan Yu, Meiyi He, Junjie Xiang, Xiao Ran, Jinyu Wu, Jun Li, Bingchuan Zhang, Amrita Chakraborty, Shulin He

**Affiliations:** 1 College of Life Sciences, Chongqing Normal University, Chongqing 401331, China; 2 Chongqing Key Laboratory of Vector Control and Utilization, Institute of Entomology and Molecular Biology, Chongqing Normal University, Chongqing 401331, China; 3 College of Environment and Ecology, Chongqing University, Shabeijie Str. 83, Chongqing 400045, China; 4 Faculty of Forestry and Wood Sciences, Czech University of Life Sciences Prague, 16500 Prague, Czech Republic

**Keywords:** caste, gut bacteriome, protist, community structure, functional prediction

## Abstract

*Coptotermes formosanus* is an economically significant termite species with a highly organised caste system, in which division of labour underpins colony function. Although gut microbiota is widely recognised for its roles in host nutrition and adaptation, much less is known about how these microbial communities are structured across castes. To explore caste-related gut bacterial variation in *C. formosanus*, we analysed the community structure of both workers and soldiers using high-throughput amplicon sequencing targeting the bacterial 16S rRNA gene. Although both castes were dominated by Bacteroidota and Spirochaetota, which together accounted for 78.87% to 85.78% in workers and 63.31% to 84.38% in soldiers, significant caste-associated differences were evident. Workers showed significantly higher bacterial richness, as indicated by observed ASVs, Chao1 indices, and Faith’s PD. Further clear caste-associated bacterial community was revealed by beta-diversity analysis. Differential taxonomic analysis revealed distinct caste-associated enrichment patterns. In addition, co-occurrence network analysis indicated a caste-associated interaction structure, with soldier-biased taxa forming a dense, highly connected module while worker-biased taxa contributed to local structure and bridging positions. Furthermore, chemoheterotrophy and fermentation were predicted to be enriched in workers, whereas nitrate reduction, aerobic chemoheterotrophy, aromatic compound degradation, and phenotypes related to biofilm formation, stress tolerance, and mobile elements were predicted to be relatively enriched in soldiers. Moreover, qPCR analysis further showed caste-associated differences in dominant gut protists, with *Pseudotrichonympha* in workers significantly higher than in soldiers and positively correlated with *Azobacteroides*. These results provide clear evidence of caste-associated differentiation in gut microbial composition and offer a foundation for identifying novel microbial targets for termite pest management.

## 1. Introduction

As primary decomposers of lignocellulose in terrestrial ecosystems, termites play a critical role in driving the global carbon cycle [1,2], but also represent a globally destructive group of economic pests. Approximately 300 of the more than 3000 known termite species are economically important pests that damage wooden structures, agroforestry systems, and timber products [3]. Termite infestations cause substantial economic losses reaching billions of dollars annually worldwide [4]. Among these pests, the invasive Formosan subterranean termite, *Coptotermes formosanus*, is particularly destructive, attacking more than 50 species of living plants as well as structural lumber [5]. Its destructive capacity is supported by an efficient gut symbiotic system for lignocellulose degradation, which enables sustained wood consumption and extensive damage to wooden structures and timber products [5,6].

The termite gut functions as a natural bioreactor for lignocellulose degradation and is characterised by efficient anaerobic fermentation [6]. The hindgut, as the main fermentation chamber in termites, harbours dense and specialized microbial communities, which interact with protozoa in lower termites, to mediate the hydrolysis and fermentation of cellulose and hemicellulose. The termite gut microbiota plays an important role in host nutrition, digestion, and overall colony functioning, and caste differentiation in termites may shape gut microbial dynamics [7,8]. In addition, gut microbiota also help compensate for the nitrogen-deficient wood feeding materials through pathways such as nitrogen fixation, uric acid cycling, and ammonia assimilation, thereby providing sustained carbon and nitrogen sources to the host [6,9]. Previous studies have shown that the composition and functional profiles of termite gut microbiota vary across termite lineages, colonies, seasons, gut regions, and environmental conditions, and are closely associated with host behaviour, digestive physiology, and social organisation [10,11,12].

The ecological success of termites also relies on the reproductive division of labour in group living. Within the colony, workers are primarily responsible for foraging and primary digestion, directly contacting and processing the wood substrate during feeding [13]. In contrast, soldiers mainly perform defensive tasks within the colony, and their energy requirements and digestive physiology differ from those of workers. Because of their limited feeding ability, soldiers rely largely on trophallaxis from workers for nutrients [8,13].

These caste-specific differences in feeding strategy and physiology are likely to influence the composition and functional potential of the gut microbiota [8]. Previous studies have shown that termite castes differ in microbial diversity, community composition, interaction networks, and predicted metabolic functions [14,15,16]. Different castes with specialized tasks in social insects were potentially associated with distinct taxonomic compositions and metabolic potentials of the gut microbiota [17,18]. For example, the gut microbiota of worker bees and drones differ significantly in dominant taxa, with worker bees enriched in bacterial groups involved in carbohydrate metabolism and drones exhibiting lower microbial diversity and reduced predicted functional capacity [19]. Similarly, in ants, gut microbial community structure was also associated with dietary sources and division of labour [20,21]. However, in termites, systematic analyses integrating caste-associated gut bacterial community structure and predicted metabolic potential between workers and soldiers of *C. formosanus* remain limited.

Recent studies have shown that *C. formosanus* harbours a defined protist assemblage including *Pseudotrichonympha*, *Holomastigotoides*, and *Cononympha*, and that different protist taxa may contribute differently to lignocellulose digestion [22,23]. The gut bacterial taxa in *C. formosanus* have been characterised and examined in several studies. For instance, more than 200 bacterial ribotypes were identified across multiple phyla, including Bacteroidota, Bacillota, and Pseudomonadota, and the overall community structure and diversity remained largely consistent between native and introduced populations [24]. In addition, caste-associated variation in gut microbiota has been documented in the wood-feeding termite *Globitermes sulphureus* [25]. Previous work using DGGE and 16S rRNA gene clone library showed that the gut bacterial communities of workers and soldiers of *C. formosanus* differed in phylogenetic composition and diversity [15]. This study identified a limited number of phylotypes and thus provided only a limited view of caste-associated gut microbial diversity and predicted functional profiles due to the low-resolution molecular approaches [15,26]. Although cross-species and multi-caste comparisons suggest that caste differentiation can influence microbial composition and certain metabolic functions in termites [26,27], these findings are largely derived from higher termites or species with different ecological strategies, limiting their relevance to *Coptotermes*. While the metabolic functions of termite gut microbiota are conserved, caste-specific divergence may occur in certain functional pathways [28]. Nevertheless, a comprehensive comparison of workers and soldiers in *C. formosanus* using high-throughput sequencing has remained lacking.

In this study, we hypothesized that, within the colony examined, workers and soldiers would differ in gut bacterial diversity and predicted functional profiles. We used high-throughput 16S rRNA gene sequencing and qPCR quantification, combined with bioinformatic and predicted functional profiles approaches, to provide a fine-scale comparison of caste-associated variation in the gut bacteriome of *C. formosanus*. By comparing workers and soldiers, we characterised and compared taxonomic composition, bacterial diversity, community structure, co-occurrence patterns, and predicted functional profiles between castes. We further quantified three dominant gut protist genera and assessed their correlations with selected bacterial genera to determine whether caste-associated bacterial variation was accompanied by changes in the broader protist–bacteria symbiotic system.

## 2. Results

### 2.1. Termite Species Verification and Gut Microbial Sequencing Statistics

By sequencing the COII of the single colony, we searched against the NCBI database using BLAST and confirmed that it was *C. formosanus* with 99.46% sequence identity to the COII of *C. formosanus* (KJ918308.1).

In total, we retrieved 701,697 raw sequences in the gut of *C. formosanus* workers and soldiers (Table 1). After quality filtering and downstream processing, 245,926 and 225,254 non-singleton sequences were retained in workers (68.50% of raw sequences) and soldiers (66.46% of raw sequences), respectively. After DADA2 denoising and ASV inference, a total of 8592 amplicon sequence variants (ASVs) were identified, including 5360 ASVs in workers and 3902 ASVs in soldiers. Taxonomic annotation (Table 2) assigned these ASVs to 19 phyla, 29 classes, 70 orders, 132 families, and 231 genera. At the caste level, ASVs from workers were classified into 17 phyla, 25 classes, 63 orders, 115 families, and 190 genera, whereas ASVs from soldiers were classified into 17 phyla, 25 classes, 53 orders, 92 families, and 151 genera.

**Figure 1:**
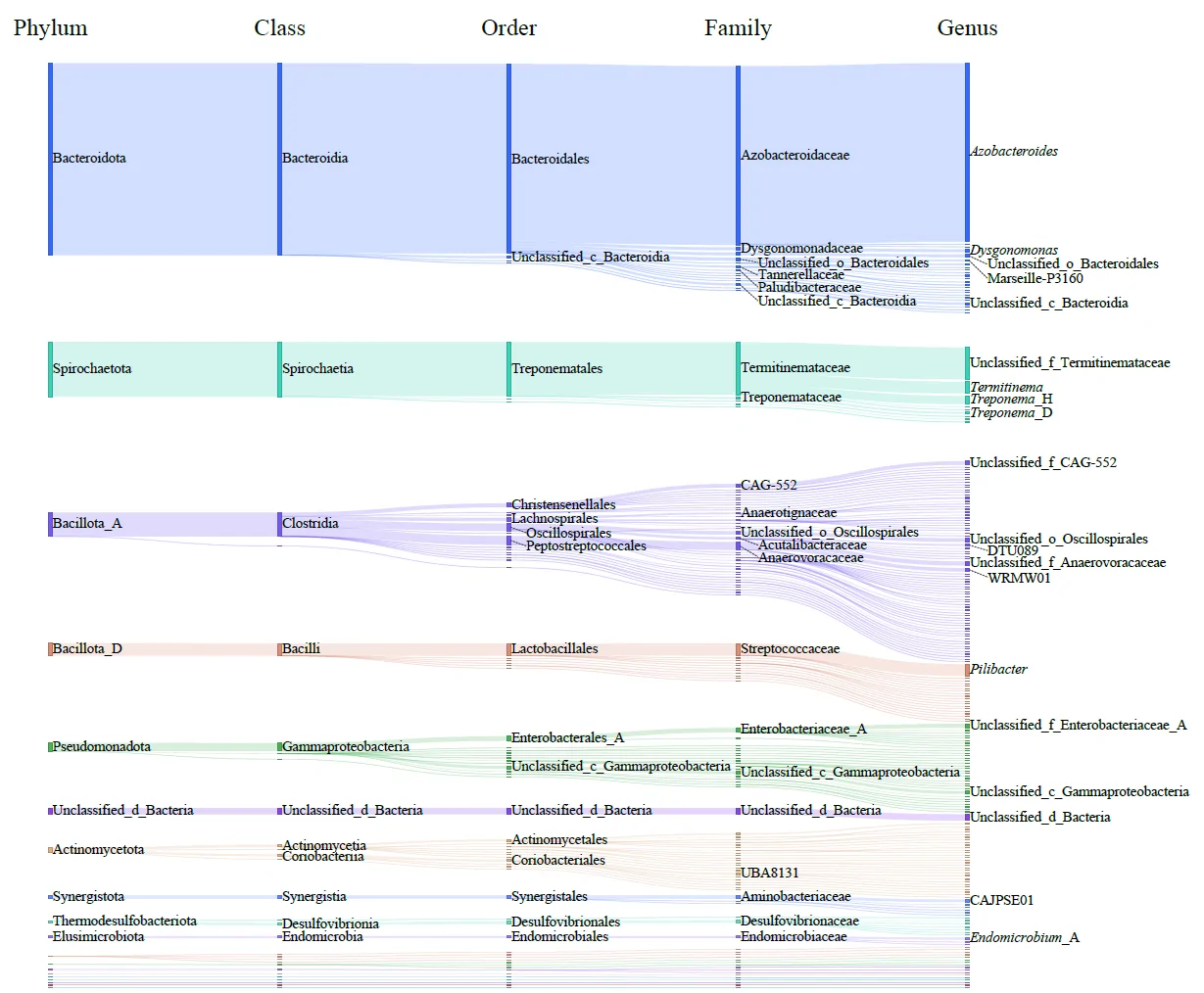
Sankey diagram illustrating the taxonomic hierarchical structure of the gut microbial community from phylum to genus, based on relative abundance ranking. The diagram highlights the major taxonomic flows within the community, with branch widths proportional to the relative abundance of each taxon.

The taxonomic hierarchical Sankey diagram based on relative abundance (Figure 1) illustrated the overall structure of the gut microbial community from phylum to genus level. It clearly shows that a few dominant lineages extended across multiple taxonomic levels and formed continuous major branches. Among these, the phylum Bacteroidota and Spirochaetota were the most prominent. In contrast, lineages within Bacillota exhibited more variable structures. The Bacillota_D branch followed a relatively concentrated pathway, whereas Bacillota_A displayed a more complex pattern, diverging into multiple parallel branches. The Pseudomonadota lineage followed a narrower pathway with a lower overall abundance. Some smaller branches were also observed, including Actinomycetota, Thermodesulfobacteriota, and uncultured bacteria.

### 2.2. Gut Community Composition in Workers and Soldiers

**Figure 2:**
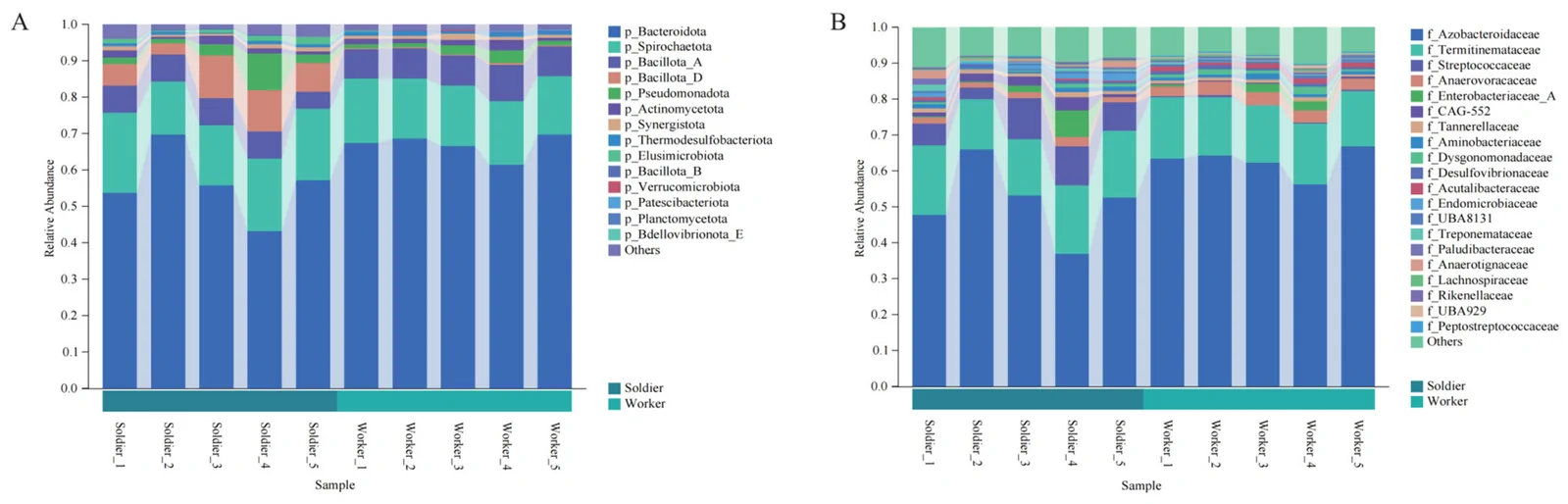
Taxonomic composition of gut microbial communities in workers and soldiers of *C. formosanus*. Relative abundance of microbial taxa at the phylum (**A**) and family (**B**) levels reflects the overall community structure and caste-associated differences.

At the phylum level, the gut microbial communities of workers and soldiers were mainly composed of Bacteroidota and Spirochaetota, with Bacteroidota being the most abundant across all samples (Figure 2A). The relative abundance of Bacteroidota ranged from 61.39% to 69.78% in workers and from 43.13% to 69.82% in soldiers. Spirochaetota accounted for 16.65% to 17.88% in workers and 14.56% to 21.89% in soldiers. The relative abundance of these dominant phyla showed greater variation in soldier samples. The relative abundance of Bacillota_A ranged in relative abundance from 7.90% to 10.01% in workers and from 4.55% to 7.50% in soldiers, showing higher abundance in workers. In contrast, Bacillota_D ranged in relative abundance from 0.13% to 0.55% in workers and from 3.02% to 11.74% in soldiers, with higher abundance in soldiers. Other phyla, including Pseudomonadota, Actinomycetota, Synergistota, and Elusimicrobiota, were present at low relative abundances in both groups, each accounting for less than 5%.

At the family level, the gut microbial communities of workers and soldiers were dominated by Azobacteroidaceae (Bacteroidota), with Azobacteroidaceae being the most abundant across all samples (Figure 2B). The relative abundance of Azobacteroidaceae ranged from 56.28% to 66.83% in workers and from 36.82% to 65.99% in soldiers, with greater variation in soldier samples. The second most abundant family was Termitinemataceae (Spirochaetota), accounting for 15.50% to 17.19% in workers and 14.05% to 19.58% in soldiers, with greater variation in soldier samples. Among Bacillota-associated lineages, Streptococcaceae (Bacillota_D) ranged in relative abundance from 0.08% to 0.50% in workers and from 2.99% to 11.47% in soldiers, with significantly higher abundance in soldiers. In contrast, Anaerovoracaceae (Bacillota_A) ranged in relative abundance from 2.63% to 3.87% in workers and from 1.26% to 2.42% in soldiers, with higher abundance in workers. Other families, including Enterobacteriaceae_A, Tannerellaceae, Aminobacteriaceae, Dysgonomonadaceae, and Desulfovibrionaceae, were present at low relative abundances in both groups.

### 2.3. The Diversity of Gut Bacterial Communities in Workers and Soldiers

**Figure 3:**
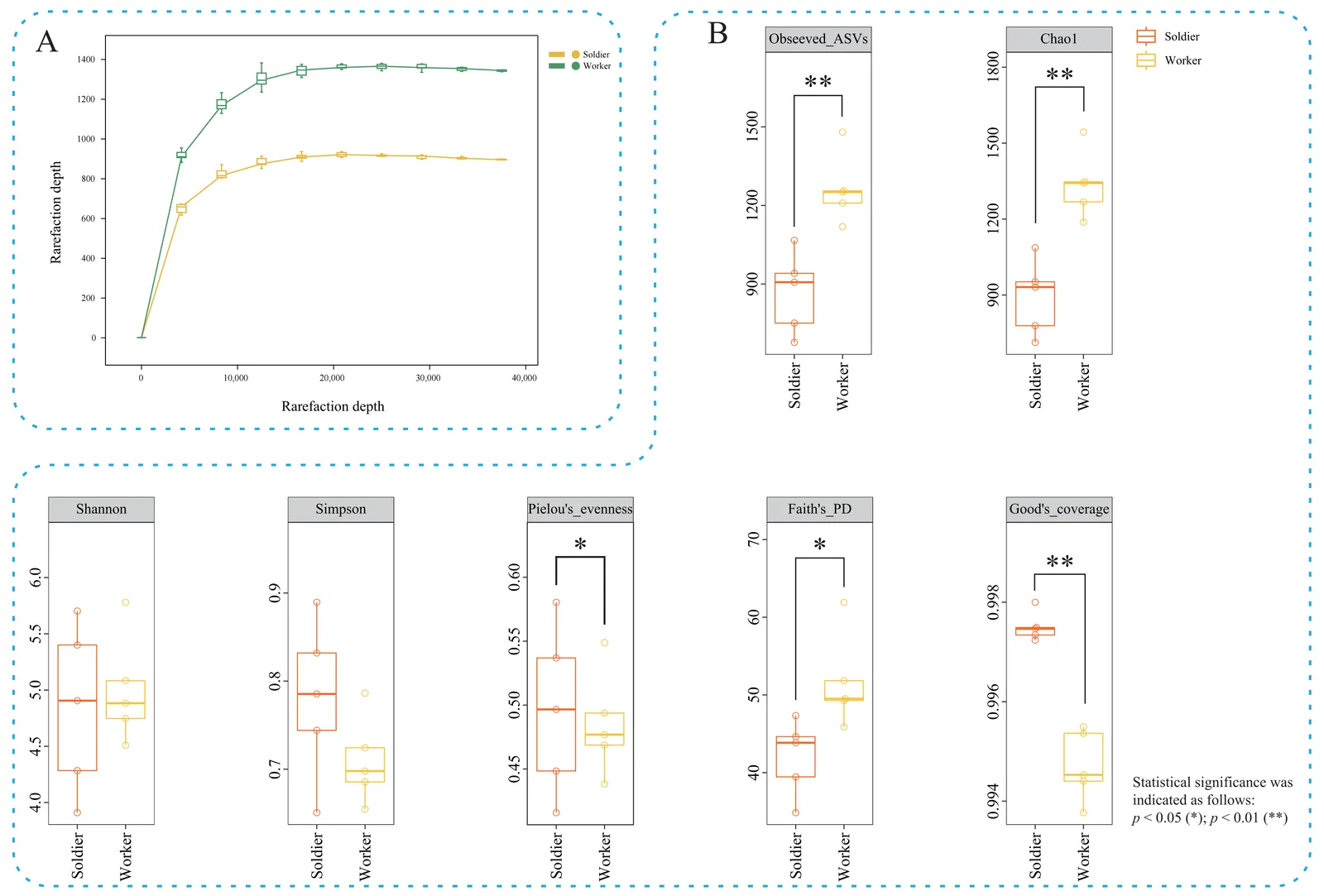
Alpha diversity and sequencing depth of gut microbial communities in workers and soldiers. Rarefaction curves (**A**) show sequencing depth and sampling completeness. Boxplots of alpha diversity indices (**B**) including richness, diversity, evenness, Faith’s phylogenetic diversity, and coverage, compare workers and soldiers.

The rarefaction curves in both groups reached plateaus (Figure 3A), indicating that the sequencing depth was sufficient to capture the diversity of most bacteria. The curve of workers reached a higher plateau than that of soldiers, indicating higher species richness in workers.

At the alpha diversity level, the species richness index of workers was generally higher than that of soldiers (Figure 3B). Both the observed ASVs index (*p* = 0.0079) and the Chao1 index (*p* = 0.0079) showed significant differences between the groups, with workers showing higher richness and a broader distribution, whereas soldiers exhibited lower richness and samples tightly clustered. However, there were no significant differences in the overall diversity and evenness between the groups, including the Shannon index (*p* = 0.8400), Simpson index (*p* = 0.3100), and Pielou’s evenness (*p* = 0.8400). Although slight variations in dispersion were observed, with a wider spread in soldiers for Shannon and Simpson, the index values were comparable between the groups, indicating comparable within-sample diversity patterns related to dominance and evenness. In contrast, significant differences in Faith’s phylogenetic diversity (*p* = 0.0160) were observed between groups, with workers showing higher values than soldiers. The Good’s coverage values were close to 1 in both castes, with soldiers showing slightly higher values and lower variability (*p* = 0.0079). This suggests marginally greater sequencing coverage in soldiers, although the small absolute difference indicates similarly high community sampling completeness in both castes.

**Figure 4:**
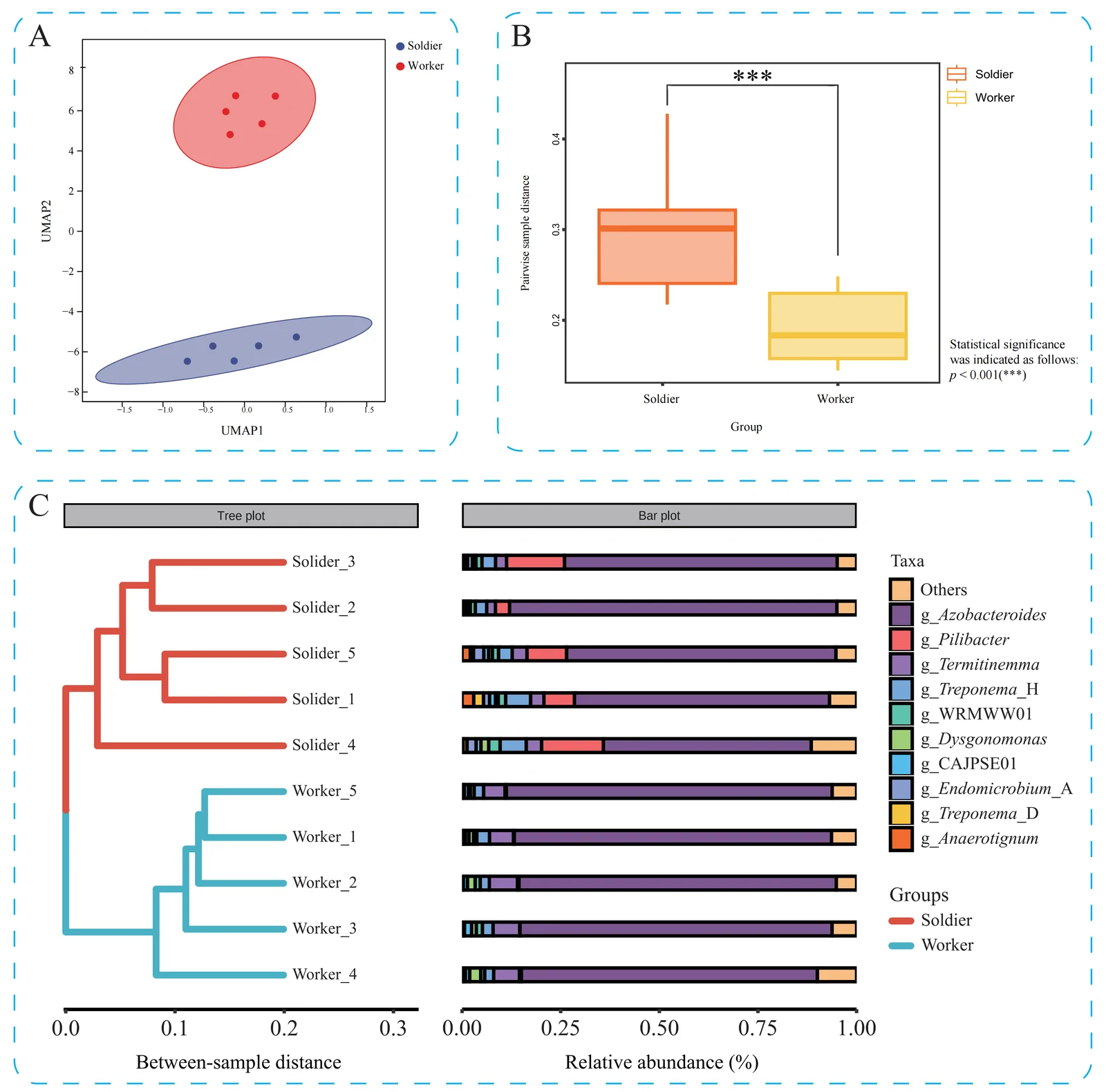
Beta diversity and community structure of gut microbial communities in workers and soldiers. UMAP ordination based on Bray–Curtis dissimilarity (**A**), showing obvious separation of workers and soldiers, with the ellipses represent 95% confidence intervals around the centroid of each group. PERMANOVA analysis with 999 permutations (**B**) shows that caste identity has a significant impact on community composition. UPGMA clustering (**C**) illustrates differences in community similarity among samples.

In the UMAP ordination, the worker and soldier samples formed two distinct clusters, indicating differences in gut bacterial community composition between castes (Figure 4A). PERMANOVA based on Bray–Curtis dissimilarity and 999 permutations confirmed significant between-caste differences in overall bacterial community composition, with caste identity explaining a substantial proportion of community variation (R^2^ = 0.514, *p* = 0.004; Figure 4B). Within-group dispersion did not differ significantly between groups (PERMDISP, *p* = 0.066).

UPGMA hierarchical clustering based on Bray–Curtis distance (Figure 4C) showed that workers and soldiers formed two distinct major branches at the genus level according to relative abundance, indicating clear differentiation in overall community composition between the two castes. The gut microbiota in all samples was mainly composed of *Azobacteroides*, along with *Termitinema*, *Treponema*_H, and WRMW01, constituting the shared core genera of both castes. Although a shared core composition was observed, differences in the relative abundance distributions of several genera were evident between groups. Specifically, *Pilibacter*, *Endomicrobium*_A, and *Anaerotignum* showed higher relative abundances in soldiers than in workers, contributing to caste-specific compositional divergence. The relative abundance of *Azobacteroides* was highly consistent in the workers. However, the abundances of *Termitinema*, *Treponema*_H, WRMW01, and *Endomicrobium*_A showed limited variation across the samples. In contrast, *Dysgonomonas*, *Pilibacter*, and CAJPSE01 remained consistently at low abundance across the samples, and maintained a stable and uniform Community membership at the genus level. Although *Azobacteroides* remained the dominant genus, the relative abundances of *Treponema*_D and *Anaerotignum* varied substantially among samples, leading to the formation of multiple sub-branches in the clustering tree.

### 2.4. Identification of Caste-Associated Differential Bacterial Taxa

**Figure 5:**
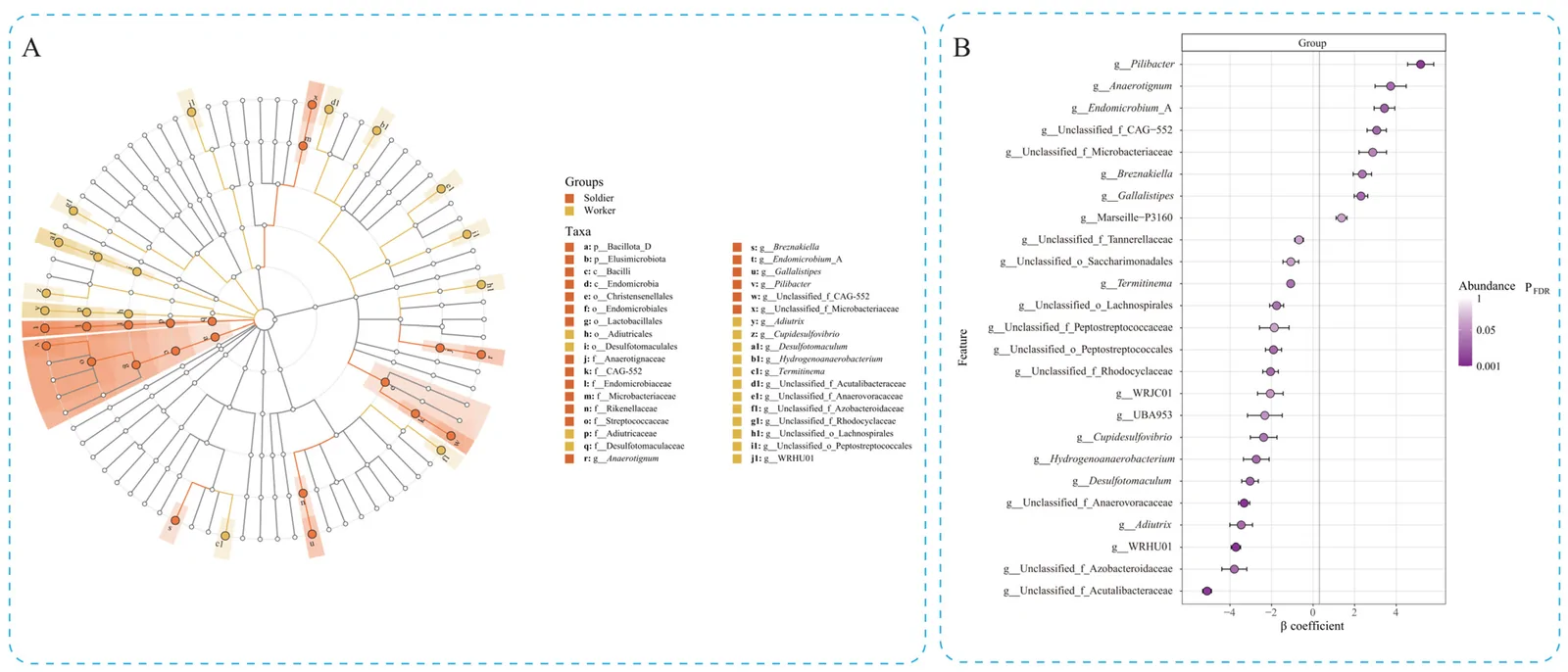
Caste-associated bacterial taxa identified using MaAsLin3. The cladogram shows significant taxa from the phylum to genus levels (**A**), with orange indicating soldier-associated taxa and yellow indicating worker-associated taxa. The genus-level coefficient plot shows MaAsLin3 abundance-model estimates and standard errors (**B**). Workers were used as the reference group; positive coefficients indicate higher abundance in soldiers, whereas negative coefficients indicate higher abundance in workers.

MaAsLin3 analysis (Figure 5A) identified 36 caste-associated bacterial taxa, comprising two phyla, two classes, five orders, eight families, and 19 genera (Figure 5A). Of these, 20 taxa showed higher non-zero relative abundances in soldiers, whereas 16 showed higher non-zero relative abundances in workers. The soldier-associated taxa included the continuous form Bacillota_D to *Pilibacter* and from Elusimicrobiota to *Endomicrobium*_A lineages. In contrast, the worker-associated taxa included from Adiutricale to *Adiutrix* and from Desulfotomaculales to *Desulfotomaculum* lineages, together with several additional genus-level taxa.

At the genus level, 19 taxa showed significant associations with caste (Figure 5B). 7 genera were positively associated with soldiers, including *Pilibacter*, Anaerotignum, *Endomicrobium*_A, *Breznakiella*, and *Gallalistipes*, as well as unclassified genera assigned to CAG-552 and Microbacteriaceae. 12 genera were negatively associated with soldiers and therefore showed higher non-zero relative abundances in workers, including *Adiutrix*, *Cupidesulfovibrio*, *Desulfotomaculum*, *Hydrogenoanaerobacterium*, *Termitinema*, and WRHU01, together with unclassified genera assigned to Acutalibacteraceae, Anaerovoracaceae, Azobacteroidaceae, Rhodocyclaceae, Lachnospirales, and Peptostreptococcales. Among the displayed associations, *Pilibacter* showed the largest positive coefficient, whereas the unclassified genus assigned to Acutalibacteraceae showed the largest negative coefficient.

### 2.5. Co-Occurrence Network Structure of Gut Microbial Communities

**Figure 6:**
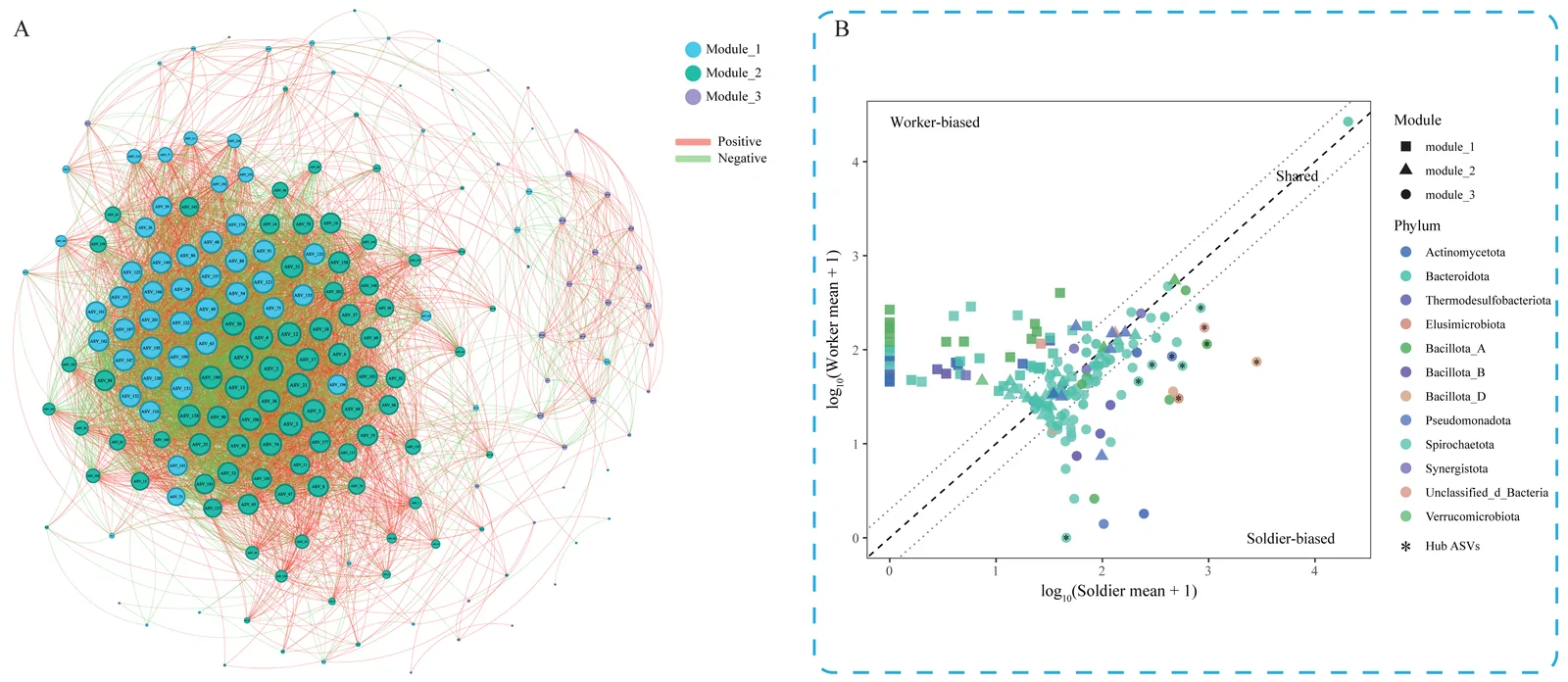
Co-occurrence network and abundance distribution of gut microbial communities in *C. formosanus*. Co-occurrence network (**A**) shows statistical co-occurrence associations among ASVs, with edges representing significant correlations (|*r*| ≥ 0.7, *p* < 0.05) and node colours indicating phylum-level taxonomy. Scatter plot (**B**) shows the relationship between mean abundances of ASVs in workers and soldiers. The shaded central region (−1 < log2 fold change < 1) represents shared taxa, while points above and below this region indicate worker- and soldier-biased taxa, respectively.

Co-occurrence network analysis was performed at the ASV level to resolve the internal interaction co-occurrence patterns of gut microbial communities (Figure 6A). Under the correlation threshold of *r* = 0.7, a total of 5487 significant co-occurrence associations (edges) were screened out, which connected 190 ASVs (nodes) and constructed a complex microbial co-occurrence network. This network displayed a mean degree of 57.76, a graph density of 0.306, a high average clustering coefficient (0.733), and a short average path length (2.203). The network was divided into three modules with 90 nodes in Module_3, 67 in Module_1, and 33 in Module_2. Based on caste bias, 76 nodes were classified as shared, 71 as worker-biased, and 43 as soldier-biased.

Most highly connected nodes were concentrated in Module_3 and were primarily soldier-biased (Figure 6B). The node with the highest degree was ASV_2 (Bacillota_D), with a degree of 114 and mean abundances of 73.6 and 2832.4 in workers and soldiers. Other prominent soldier-biased hubs included ASV_3 (Spirochaetota), ASV_9 (Spirochaetota), and ASV_21 (Spirochaetota), each with a degree of 112, as well as ASV_12 (Elusimicrobiota), corresponding to *Endomicrobium*_A (degree = 111). Conversely, nodes in Module_1 were largely worker-biased. Among these, ASV_29 (Bacillota_A), classified as Anaerovoracaceae, exhibited a degree of 104. Module_2 had fewer nodes and lacked a highly connected core comparable to those of Module_3 and Module_1.

Closer inspection of local connectivity patterns revealed that ASV_12 (Elusimicrobiota, soldier-biased) formed associations with 111 nodes, comprising 68 positive and 43 negative interactions. Its strongest connections were observed with ASV_21 (Spirochaetota, soldier-biased) (*r* = 0.9710), ASV_2 (Bacillota_D, soldier-biased) (*r* = 0.9668), ASV_4 (Unclassified_d_Bacteria, soldier-biased) (*r* = 0.9594), and ASV_9 (Spirochaetota, soldier-biased) (*r* = 0.9531). These highly connected soldier-biased nodes together formed a dense module within the network. Among the worker-biased nodes, ASV_29 (Bacillota_A, worker-biased) showed strong positive correlations with several worker-biased nodes, including ASV_48 (Bacillota_A, worker-biased) (*r* = 0.9313), ASV_49 (Unclassified_d_Bacteria, worker-biased) (*r* = 0.9262), and ASV_54 (Bacillota_A, worker-biased) (*r* = 0.9169). In contrast, ASV_29 (Bacillota_A, worker-biased) showed strong negative correlations with ASV_12 (Elusimicrobiota, soldier-biased) (*r* = −0.9098) and ASV_2 (Bacillota_D, soldier-biased) (*r* = −0.9027).

In addition to high-degree nodes, betweenness centrality analysis identified a group of nodes with potential bridging roles. The highest betweenness was observed for ASV_8 (Spirochaetota, Shared), followed by ASV_171 (Bacteroidota, worker-biased), ASV_34 (Bacteroidota, worker-biased), ASV_26 (Spirochaetota, worker-biased), ASV_12 (Elusimicrobiota, soldier-biased), and ASV_49 (Unclassified_d_Bacteria, worker-biased). These nodes showed relatively high importance in linking different local structures within the network.

### 2.6. Predicted Functional Profiles of Gut Bacterial Communities in Workers and Soldiers

**Figure 7:**
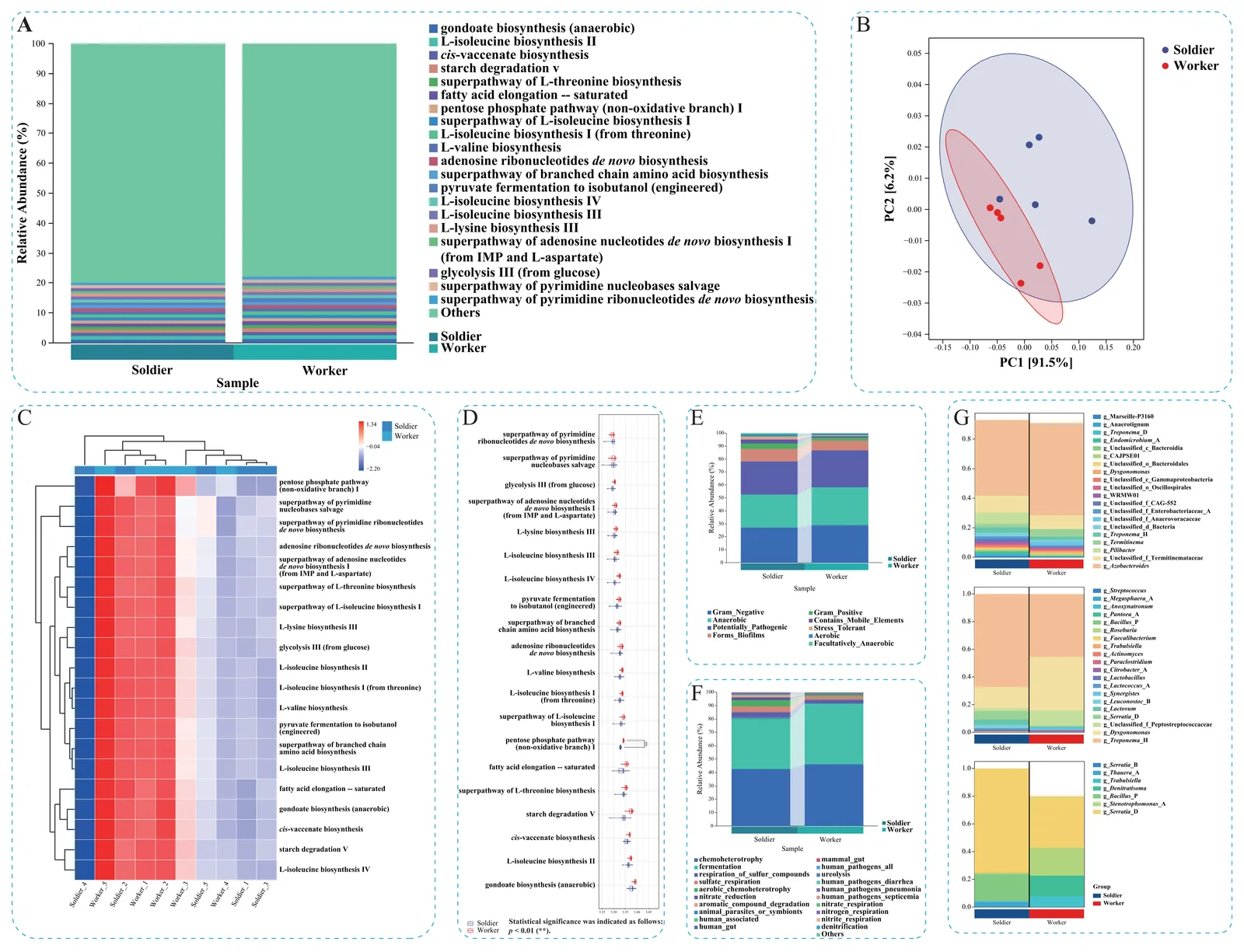
Functional prediction of termite gut microbiota in *C. formosanus* based on PICRUSt2 and complementary annotation tools. Relative abundance of predicted MetaCyc pathways (**A**) shows functional differences between workers and soldiers. Principal coordinates analysis (PCoA) (**B**) illustrates separation patterns based on pathway composition. Hierarchical clustering heatmap (**C**) presents Z-score normalized pathway abundance, highlighting both sample clustering and pathway grouping patterns. Differential pathway analysis (**D**) identifies significantly enriched pathways between groups based on Wilcoxon test. Phenotypic prediction (**E**) based on BugBase reveals differences in microbial traits. Functional annotation (**F**) using FAPROTAX indicates dominant ecological functions among the taxonomically annotated fraction of the bacterial community. Genus-level contribution analysis (**G**) highlights key taxa involved in selected functions, including pentose phosphate pathway (non-oxidative branch), fermentation, and nitrate reduction pathway.

The predicted functional profiles of the termite gut bacterial community were inferred using PICRUSt2 and analysed at the MetaCyc pathway level. Because these functional profiles were inferred from 16S rRNA gene data, the following results represent predicted metabolic potential rather than direct evidence of microbial gene expression or metabolic activity. The weighted NSTI values for all samples ranged from 0.184 to 0.263, with mean values of 0.224 for soldiers and 0.198 for workers. The major functional pathways in MetaCyc (Figure 7A) were associated with amino acid metabolism (L-isoleucine biosynthesis II, pathway of L-threonine biosynthesis, L-isoleucine biosynthesis I [from threonine], and L-valine biosynthesis), lipid metabolism (gondoate biosynthesis [anaerobic], cis-vaccenate biosynthesis, and saturated fatty acid elongation), carbohydrate metabolism (starch degradation V), and branched-chain amino acid biosynthesis (pathway of branched chain amino acid biosynthesis). The functional composition differed only slightly between groups. The soldiers and workers showed largely similar trends in the relative abundance distribution of each pathway, with minor fluctuations in some pathways. PCoA analysis (Figure 7B) showed that PC1 and PC2 explained 91.5% and 6.2% of the variance, respectively.

At the functional pathway level (Figure 7C), 20 pathways were divided into two major functional clusters. Among them, the pentose phosphate pathway (non-oxidative branch) formed an independent branch by itself. The remaining pathways constituted the second major cluster and were further subdivided. The first subcluster consisted of the pathway of pyrimidine nucleobases salvage and the pathway of pyrimidine ribonucleotides de novo biosynthesis, whereas the remaining pathways belonged to a more complex branch, which was further divided into two modules. The first module mainly included pathways related to amino acid biosynthesis and carbon metabolism, including adenosine ribonucleotides de novo biosynthesis, pathway of adenosine nucleotides de novo biosynthesis I (from IMP and L-aspartate), pathway of L-threonine biosynthesis, pathway of L-isoleucine biosynthesis I, L-lysine biosynthesis III, glycolysis III (from glucose), L-isoleucine biosynthesis II, L-isoleucine biosynthesis I (from threonine), L-valine biosynthesis, pyruvate fermentation to isobutanol (engineered), pathway of branched chain amino acid biosynthesis, and L-isoleucine biosynthesis III. The second module mainly included pathways related to fatty acid metabolism and some core metabolic processes, including saturated fatty acid elongation, gondoate biosynthesis (anaerobic), *cis*-vaccenate biosynthesis, starch degradation V, and L-isoleucine biosynthesis IV. Differential pathway analysis (D) showed that only the pentose phosphate pathway (non-oxidative branch) differed significantly between workers and soldiers. The remaining predicted MetaCyc pathways did not show significant caste-associated differences, supporting the view that workers and soldiers shared a largely conserved core functional profile.

Based on BugBase predictions (Figure 7E), differences in predicted phenotype composition were identified between the soldiers and workers. Gram-negative, anaerobic, and potentially pathogenic phenotypes were predicted to account for relatively high proportions in both groups, and all three were slightly higher in the workers than in the soldiers. In contrast, the biofilms and Gram-positive phenotypes were predicted to be relatively more represented in the soldiers. In addition, the mobile elements and stress tolerant phenotypes were predicted at relatively higher levels in the soldiers.

FAPROTAX-based annotation predicted (Figure 7F) chemoheterotrophy and fermentation as the dominant ecological functions of the gut microbial community. Both functions were predominant in the two groups, with relatively higher predicted representation in the workers. Among other functional categories, aerobic chemoheterotrophy, nitrate reduction, and aromatic compound degradation were predicted to be relatively more represented in the soldiers. For example, the predicted relative abundance of nitrate reduction was approximately 2.17% in the soldiers but only approximately 0.26% in the workers. In addition, some predicted functions related to nitrogen cycling showed generally low abundance but still differed between the two groups. For example, denitrification was absent in the soldiers, whereas it was slightly higher in the workers than in the soldiers. Predicted nitrite respiration was also relatively higher in the workers than in the soldiers.

Predicted genus-level contributions (Figure 7G) to key pathways and ecological functions were further analysed. The results showed that in the pentose phosphate pathway (non-oxidative branch) pathway, the predicted pathway contribution was mainly attributable to taxa related to *Azobacteroides*, and unclassified_f_Termitinemataceae in both castes. *Azobacteroides* was predicted to contribute a higher proportion in workers, whereas *Pilibacter*, *Treponema*_H, *Endomicrobium*_A, and several unclassified bacterial taxa were predicted to contribute relatively higher proportions in soldiers. For fermentation related functions, the predicted contribution was mainly derived from *Treponema*_H and *Dysgonomonas* in both castes. *Treponema*_H, *Serratia*_D, *Lactovum*, and *Leuconostoc*_B were predicted to contribute higher proportions in soldiers, whereas *Dysgonomonas* and unclassified_f_Peptostreptococcaceae were predicted to contribute higher proportions in workers. For nitrogen cycling-related functions, such as nitrate reduction, the major predicted contributor was *Serratia*_D, particularly in soldiers. *Bacillus*_P and *Thauera*_A also were predicted to contribute to nitrate reduction in soldiers, whereas *Stenotrophomonas*_A, *Denitratisoma*, and *Trabulsiella* contributed relatively higher proportions in workers.

### 2.7. Caste-Associated Protist Abundance and Correlations with Selected Bacterial Genera

**Figure 8:**
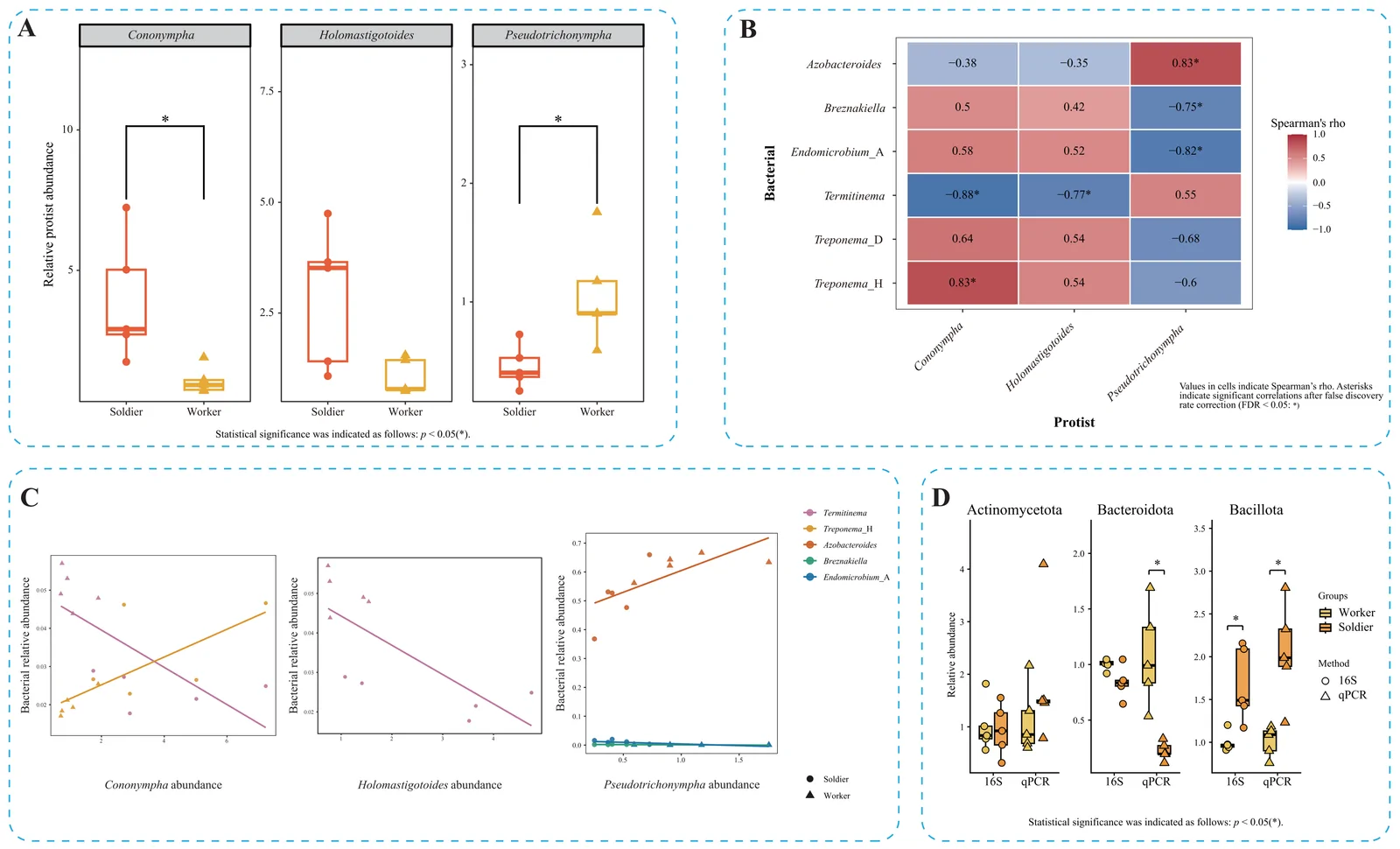
QPCR-based relative protist abundance, protist–bacteria associations and comparison of selected bacterial phyla in the gut of workers and soldiers. QPCR analysis (**A**) was performed to quantify the relative abundance of three dominant gut protist genera using the 2^−ΔΔCt^ method. Spearman’s rank correlation analysis (**B**) was conducted between protist abundance and the relative abundance of selected bacterial genera, with values in cells indicating Spearman’s *rho* and asterisks indicating FDR < 0.05. The scatterplots (**C**) show representative significant associations between protist abundance and bacterial relative abundance across matched biological replicates. Colors indicate bacterial genera, and shapes indicate caste, with circles representing soldiers and triangles representing workers. The relative abundances of three selected bacterial phyla determined by 16S rRNA gene sequencing and qPCR were compared between workers and soldiers (**D**). Colors indicate caste, with yellow representing workers and orange representing soldiers, whereas shapes indicate the detection method, with circles representing 16S rRNA gene sequencing and triangles representing qPCR.

The relative abundance of three dominant gut protist genera was quantified by qPCR and compared between workers and soldiers (Figure 8A). *Cononympha* was significantly more abundant in soldiers, whereas *Pseudotrichonympha* was significantly more abundant in workers after multiple-testing correction. *Holomastigotoides* showed a higher median abundance in soldiers, but the difference was not statistically significant.

Spearman’s rank correlation analysis was performed between protist relative abundance and the abundance of six selected bacterial genera (Figure 8B). Among the 18 tested combinations, six correlations remained significant after FDR correction. *Azobacteroides* was positively correlated with *Pseudotrichonympha* abundance (*rho* = 0.8303, FDR = 0.0172), and *Treponema*_H was positively correlated with *Cononympha* abundance (*rho* = 0.8303, FDR = 0.0172). In contrast, *Termitinema* was negatively correlated with both *Cononympha* (*rho* = −0.8788, FDR = 0.0146) and *Holomastigotoides* (*rho* = −0.7697, FDR = 0.0332). Negative correlations were also observed between *Endomicrobium*_A and *Pseudotrichonympha* (*rho* = −0.8182, FDR = 0.0172), and between *Breznakiella* and *Pseudotrichonympha* (*rho* = −0.7455, FDR = 0.0400). Scatterplots of the significant associations further illustrated these protist–bacteria relationships (Figure 8C).

The qPCR results (Figure 8D) showed caste-associated trends generally consistent with those obtained from 16S rRNA gene sequencing. Bacteroidota was lower in soldiers than in workers, with the difference reaching significance only in the qPCR analysis. Bacillota was significantly higher in soldiers according to both methods. No significant caste-associated difference was detected for Actinomycetota. These consistent trends further supported the reliability of the bacterial abundance patterns identified by 16S rRNA gene sequencing.

## 3. Discussion

The termite gut microbiota plays an important role in host nutrition, digestion, and colony functioning, and may be shaped by caste differentiation. Here we characterised caste-associated variation in the *C. formosanus* gut bacteriome by using high-throughput 16S rRNA gene sequencing and analyzing microbial diversity, co-occurrence network, and predicted functional profiles. The results showed that both workers and soldiers were dominated by Bacteroidota and Spirochaetota, but differed in bacterial richness, Faith’s PD, community composition, discriminatory taxa, co-occurrence patterns, and predicted functional profiles. In addition, based on qPCR protist quantification and correlation analysis indicated that several selected bacterial genera co-varied with dominant gut protists, suggesting that caste-associated bacterial shifts may occur alongside changes in dominant protist abundance. Our findings corroborate earlier reports that worker and soldier castes in *C. formosanus* harbor a stable bacteriome dominated by Bacteroidota and Spirochaetota [6,27]. These caste-associated differences have been reported in whole-gut analyses of *C. formosanus*, with workers generally harboring higher microbial diversity and exhibiting greater functional complexity than soldiers [15].

Furthermore, the functional prediction suggested that, while most central metabolic pathways remained largely conserved across castes, several metabolic pathways, ecological functions, and phenotype-associated traits differed between the castes.

At the taxonomic level, *C. formosanus* gut bacteriome showed structural adjustments within a shared community framework. Workers and soldiers were dominated by Bacteroidota and Spirochaetota; however, these dominant groups were maintained differently between castes. Workers retained Bacteroidota populations at a high and stable relative abundance, whereas soldiers showed greater fluctuations and an overall decrease in their abundance. In contrast, Spirochaetota displayed a broader range of variation in soldiers and increased in relative abundance. These shifts did not disrupt the overall community structure; instead, they reshaped the relative proportions within. These differences became clearer at finer taxonomic resolution, where bacterial genera *Azobacteroides* and *Pilibacter* in soldiers exhibited opposing abundance patterns compared with in workers. This contrasted abundance may reflect differing ecological associations; such taxa may function in complementary or substitutive ways within the same host environment, helping preserve gut bacterial stability.

Additionally, these caste-associated differences were further reflected in community structure. Beta-diversity analysis revealed distinct clustering of gut bacterial communities between workers and soldiers, indicating that caste identity was strongly associated with gut bacterial composition. A plausible explanation could be the ecological filtering associated with caste-specific feeding biology. Workers are the primary feeders and initial digesters within the colony, directly consuming and processing complex lignocellulosic substrates. Because termite gut microbiota are central to lignocellulose digestion and host nutritional adaptation, the direct involvement of workers in foraging and lignocellulose processing may increase their exposure to substrate-associated microorganisms and provide a broader range of nutritional niches for gut bacteria [5,27,29]. As a result, their gut contents likely encompass spatial and chemical heterogeneity, allowing a wider range of microbial niches to be maintained. On the contrary, soldiers depend largely on trophallaxis and therefore receive uniform, preprocessed nutrients. Such a diet would impose a stronger ecological filter, constraining niche diversity and limiting the persistence of additional microbial lineages [9,30]. This pattern is consistent with previous studies, which showed higher microbial complexity in workers and a relative increase in Spirochaetota in soldiers [15].

This taxonomic difference was highly consistent with known functional specialization in the termite gut system. Bacteroidota-related taxa typically participate in lignocellulose degradation, nitrogen metabolism, and amino acid synthesis, and play a key role in digestion and nutrient acquisition during active feeding by workers [8,27]. In contrast, Bacillota-related taxa participate in the utilization and transformation of fermentation intermediates, which aligns with the lifestyle of soldiers that rely on trophallaxis for nutrient intake [31].

However, this difference did not alter the overall metabolic framework. Most predicted metabolic pathways were broadly similar between workers and soldiers, suggesting a largely conserved predicted functional framework for the fundamental digestive and fermentative functions required by the termite holobiont. The gut bacterial communities in workers were predicted to possess relatively greater functional potential for amino acid metabolism and carbon metabolism, whereas soldiers’ gut bacteriome showed relatively higher predicted representation of functions related to transport, genetic information processing, nitrate reduction, and the degradation of aromatic compounds. Additionally, phenotypic traits such as biofilm formation, Gram-positive characteristics, mobile genetic elements, and stress tolerance were predicted to be relatively more represented in soldiers. These differences were also found in other termite species supported. For instance, in *Reticulitermes flaviceps*, soldiers showed a loss of key fiber-degrading functions [8]. In *C. formosanus*, however, soldiers retained the main metabolic framework while tuned for specific functions. These results suggest that caste differentiation is accompanied by functional adjustment within a stable metabolic system, rather than by loss or replacement of functional capacity.

Notably, ecological importance was not always aligned with the taxa retained by the combined differential-abundance analysis. Several taxa that were not retained in the final biomarker set nevertheless showed high connectivity in the co-occurrence network. This indicated their ecological importance in the termite gut. Similarly, Elusimicrobiota had relatively low overall abundance but displayed strong connectivity in the network analysis, suggesting that low-abundance taxa may still act as key ecological nodes. This was further supported by predicted taxon-contribution analysis. Carbon metabolism was predicted to be mainly associated with taxa such as *Dysgonomonas*, *Lactobacillus*, and *Treponema*. *Lactobacillus* was predicted to contribute more strongly in workers, whereas *Dysgonomonas* and several unclassified anaerobes showed relatively greater predicted contributions in soldiers. Nitrogen cycle-related functions, particularly nitrate reduction, were predicted to be relatively more represented in soldiers and were predicted to be associated mainly with bacterial genera including *Serratia*, *Trabulsiella*, and *Thauera*. Though, it is worth noting that these analyses infer functional profiles from 16S rRNA gene data rather than directly measuring microbial gene expression or metabolic activity; hence, further metatranscriptomics and functional assays are required to validate these interpretations.

In addition to bacterial taxonomic differences, qPCR-based protist quantification suggested that caste-associated bacterial shifts may be linked to variation in gut protist abundance. Lower termites depend on flagellated gut protists for lignocellulose digestion, and these protists form a multilayered symbiotic system with diverse bacterial and archaeal symbionts [32,33]. In the present study, *Cononympha* and *Holomastigotoides* were more abundant in soldiers, whereas *Pseudotrichonympha* was more abundant in workers, suggesting that dominant protist genera also showed caste-associated abundance patterns. Spearman correlation analysis further showed that several selected bacterial genera co-varied with protist abundance. Among these associations, the positive correlation between *Azobacteroides* and *Pseudotrichonympha* was the most consistent and biologically meaningful. This result is consistent with previous evidence that “Candidatus *Azobacteroides* pseudotrichonymphae” is an intracellular bacterial symbiont of *Pseudotrichonympha grassii* and may contribute to nitrogen metabolism within the protist host [34]. Therefore, caste-associated variation in *Azobacteroides* may partly reflect changes in *Pseudotrichonympha* abundance rather than bacterial community variation alone.

The positive correlation between *Treponema*_H and *Cononympha* may also indicate a protist-related bacterial pattern. Termite gut spirochetes are abundant and diverse, and recent phylogenomic work has shown that many termite-associated treponemes represent specialized lineages within the termite gut ecosystem [35]. In wood-feeding, protist-dependent termites, treponemes may occur as free-living bacteria or as protist-associated lineages, and some participate in acetogenesis, nitrogen metabolism, or hydrogen turnover. Thus, the association between *Treponema*_H and *Cononympha* may reflect a shared protist-associated microhabitat rather than a simple direct host-specific relationship. In contrast, the negative correlations involving *Termitinema*, *Endomicrobium*_A, and *Breznakiella* should be interpreted cautiously. Endomicrobia are well known as endosymbionts of termite gut flagellates, but their associations may vary among different protist hosts, and recent single-cell and genomic studies have shown that these intracellular symbionts can differ in genomic and metabolic features among protist lineages [36,37]. Therefore, these negative correlations may reflect caste-associated niche partitioning, differences in hindgut microenvironmental structure, or relative shifts among different symbiotic components rather than direct antagonistic interactions.

Although this study provides insights into the caste-associated bacterial community structure in an ecologically important termite *C. formosanus*, several limitations are worth noting. In this study, we analyzed the data from one colony due to the limited availability of one large and healthy colony in the lab. The fact that all biological replicates originated from a single colony limits generalisation of the observed caste-associated differences to other lab colonies or field collected colonies. Previous studies have shown that termite gut microbiomes varied with colony background, season, dietary substrate, and rearing or environmental conditions [11,38,39,40]. However, these differences across colonies of the same termite species in fungus-growing termites were less significant compared with the difference between castes, suggesting that castes produce stronger microbial differentiation than colony background in some termite systems [26]. In addition, termite gut microbiota were also relatively stable within termite species across geographic origins or colonies [8,24,41]. Therefore, the present findings should be interpreted as caste-associated patterns within the studied colony, and additional independent colonies are required to determine whether these patterns are consistent across *C. formosanus* colonies.

The functional interpretations are based on 16S rRNA gene data, thus represent predicted metabolic potential rather than direct evidence of microbial activity. Relatively high NSTI values in PICRUSt2 indicate that a considerable proportion of the ASVs were phylogenetically distant from the microbial reference genomes available in the PICRUSt2 database; this imposes critical constraints on the reliability of the functional predictions. In addition, due to many uncultured bacterial taxa in the termite gut, the functional prediction based on FAPROTAX also had limitations as it relies explicitly on phenotypic data derived from cultured isolates. Further validation using metagenomic, metatranscriptomic, and metabolomic approaches will be necessary to confirm the pathways and ecological functions inferred here. Although qPCR-based protist quantification and Spearman correlation analysis revealed potential protist–bacteria associations, these correlations were based on relative abundance data and should not be interpreted as direct symbiosis or functional dependence. Additional validation using fluorescence in situ hybridization, protist isolation, single-cell approaches, or metatranscriptomics will be required to confirm these interactions. Expanding future studies to include multiple colonies, developmental stages, and environmental settings will be essential for testing the consistency and ecological relevance of the caste-associated patterns reported here.

In summary, this study compared the gut microbiota of workers and soldiers of *C. formosanus*. The results reflected multilevel structural reorganization within a shared system; both castes were dominated by Bacteroidota and Spirochaetota, while their bacterial communities had contrasted composition and diversity. Furthermore, the different bacterial communities differed at the predicted functional profile, mainly in pathways related to nutrient metabolism and transport. Moreover, the qPCR-based protist analysis further suggests that this caste-associated reorganisation may involve not only bacterial community shifts, but also coordinated changes between gut protists and selected bacterial symbionts. Together, these findings indicate that, within the colony examined, caste differentiation in *C. formosanus* is accompanied by differences in predicted functional profiles, providing new insight into the ecological organisation and adaptive flexibility of the termite gut symbiosis.

## 4. Materials and Methods

### 4.1. Sample Collection

To ensure that workers and soldiers shared the same colony background, diet, and rearing conditions, samples were collected from a laboratory-maintained colony of *C. formosanus* established in Shapingba District, Chongqing, China. Individuals were maintained in a controlled-environment chamber at 25 ± 1 °C and 80 ± 5% relative humidity, with moistened birch wood provided as the food substrate and moisture maintained through regular water supplementation. Species identity was confirmed by amplifying the mitochondrial cytochrome c oxidase subunit II (COII) gene region using primers A-tLeu (5′-CAGATAAGTGCATTGGATTT-3′) and B-tLys (5′-GTTTAAGAGACCAGTACTTG-3′), followed by BLAST (version 2.17.0) comparison against sequences in the NCBI database [42,43].

### 4.2. Gut Dissection and Microbial Communities Sequencing

Worker and soldier individuals were randomly selected for the experiment. Under sterile conditions, the head was removed, and the entire gut was dissected and transferred into sterile phosphate-buffered saline (PBS). The foregut was then separated and discarded, and the midgut and hindgut were collected. Gut tissues from 20 individuals were pooled in a sterile tube containing 100 µL PBS to form one biological replicate, and five replicates were prepared for each caste.

Total genomic DNA was extracted using a commercial kit (MP Biomedicals, Irvine, CA, USA) following manufacturer’s protocol and used as the template for amplification of the V3–V4 hypervariable regions of the bacterial 16S rRNA gene using the primer pair 338F (5′-ACTCCTACGGGAGGCAGCA-3′) and 806R (5′-GGACTACHVGGGTWTCTAAT-3′). PCR products were verified by 2% agarose gel electrophoresis to confirm amplification, and DNA concentrations were quantified using the Quant-iT PicoGreen dsDNA Assay Kit. Following quantification, amplicons from different samples were pooled in equimolar concentrations and purified using AMPure XP beads (Beckman Coulter, Brea, CA, USA). The purified products were then used for library construction according to the standard Illumina amplicon library preparation protocol. Paired-end sequencing (2 × 250 bp) was subsequently performed on the Illumina NovaSeq 6000 platform (Illumina, San Diego, CA, USA) to generate raw sequencing reads.

### 4.3. Microbial Communities Structure Analysis

Raw sequencing reads were processed in QIIME2 (version 2024.5). After demultiplexing with the demux and trimming primers using Cutadapt (version 4.8), we used the DADA2 (version 1.30.0) for quality filtering with truncate length at 222 and 229 for forward and reverse sequences as well as maxEE at 4 for both sequences, denoising, paired-end merging, and chimera removal with consensus method. After these steps, we obtained the amplicon sequence variant (ASV) table and representative sequences [44]. Taxonomic assignment was carried out using the SILVA database (release 138), supplemented by the Greengenes2 database. Singleton ASVs, mitochondrial sequences as well as unassigned ASVs were removed prior to constructing a relative abundance table [45]. To account for differences in sequencing depth across samples, all samples were rarefied to 39,480 sequences per sample, corresponding to the minimum sequencing depth observed. The rarefied, taxonomically annotated ASV table was used for alpha diversity, beta diversity, PERMANOVA, taxonomic composition, and MaAsLin3 analyses. Rarefaction curves were generated to assess sequencing coverage and sampling adequacy. Relative abundances at different taxonomic levels were then calculated from the rarefied table by dividing the abundance of each taxon by the total abundance of the corresponding sample and were subsequently summarized and visualised using bar plots and Sankey diagrams [46].

Alpha diversity indices, including observed ASVs, Chao1 richness, Shannon diversity, Simpson diversity, Pielou’s evenness, Faith’s PD, and Good’s coverage, were calculated to characterise within-sample diversity by QIIME2. Differences between groups were assessed using two-sided Wilcoxon rank-sum tests, with *p* < 0.05 considered statistically significant. Beta diversity was calculated using Bray–Curtis dissimilarity and community structure was determined using uniform manifold approximation and projection (UMAP). Between-caste differences in overall bacterial community composition were tested using permutational multivariate analysis of variance (PERMANOVA) based on the Bray–Curtis dissimilarity matrix with 999 permutations. To distinguish compositional separation from differences in group spread, within-group multivariate dispersion was evaluated using permutational analysis of multivariate dispersions (PERMDISP) [47].

Differential taxonomic analyses were performed using the unrarefied count tables aggregated at the phylum, class, order, family, and genus levels. Taxa with a mean relative abundance below 0.02% or detected in fewer than 20% of the samples were excluded. The MaAsLin3 non-zero abundance model was fitted separately at each taxonomic level using total-sum scaling normalization and logarithmic transformation [48]. Caste was included as the fixed effect, with workers specified as the reference group, and the median-based abundance comparison was enabled. Statistical significance was defined as a Benjamini–Hochberg-adjusted *q*-value < 0.05. Positive coefficients indicated higher non-zero relative abundance in soldiers, whereas negative coefficients indicated higher non-zero relative abundance in workers. Genus-level associations were summarized using the estimated β coefficients and their standard errors.

### 4.4. Co-Occurrence Network Analysis

Bacterial co-occurrence networks were constructed based on correlations among ASVs using SparCC (Sparse Correlations for Compositional Data) [49]. The unrarefied, taxonomically annotated ASV table was used for network analysis. ASV counts were converted to relative abundances by dividing the count of each ASV by the total sequencing depth of the corresponding sample. Pairwise correlations were calculated based on the relative abundance matrix, and pseudo-*p* values were generated using permutation testing. Only robust correlations with an absolute correlation coefficient (|*r|*) > 0.7 and *p* < 0.05 were retained for network construction. The resulting correlation matrix was used to construct the co-occurrence network. Network topological properties, including node degree and betweenness centrality, were calculated to identify key taxa within the network. Nodes with relatively high degree or betweenness centrality were considered potential keystone taxa. Network visualisation and layout optimisation were performed using Gephi (version 0.10.1) [50].

To characterise caste-associated distribution patterns, the mean relative abundance of each ASV was calculated for workers and soldiers as the average across all samples within each group (Worker_mean and Soldier_mean). A $\log_{2}\mathrm{fold}C\mathrm{hang}e$ ($\log_{2}\mathrm{FC}$) was then computed as $\log_{2}\frac{\mathrm{Worker}\_\mathrm{mean}+1\times 10^{-6}}{\mathrm{Soldier}\_\mathrm{mean}+1\times 10^{-6}}$, where a small pseudocount (1 × 10^−6^) was added to avoid division by zero. Based on $\log_{2}\mathrm{FC}$ values, ASVs were classified into three categories: worker-associated ($\log_{2}\mathrm{FC}$ > 1), soldier-associated ($\log_{2}\mathrm{FC}$ < −1), and shared (−1 ≤ $\log_{2}\mathrm{FC}$ ≤ 1).

### 4.5. Microbial Functional Prediction and Differential Functional Analysis

The predicted metabolic potential of the bacterial communities was predicted using PICRUSt2 based on 16S rRNA gene sequencing data [51], and was annotated at the MetaCyc pathway level [52]. The functional prediction was evaluated using the weighted nearest sequenced taxon index (NSTI). The relative abundance of predicted pathways was used for downstream analyses, and the 30 most abundant pathways, ranked according to their mean abundance across groups, were selected for visualization. Differences in pathway abundance between workers and soldiers were assessed using the Wilcoxon rank-sum test, and *p*-values were adjusted using the BH to control the false discovery rate. Based on pathway abundance profiles, principal coordinates analysis (PCoA) and hierarchical clustering were performed to evaluate functional structure variation and grouping patterns among samples. Microbial phenotypes, including gram staining characteristics, oxygen requirements, and potential pathogenicity, were predicted using BugBase to compare differences among groups. Ecological functions were annotated using the FAPROTAX database, with particular focus on carbon metabolism, nitrogen cycling, and organic matter degradation [53]. Furthermore, species contribution analysis was conducted to quantify the contribution of different taxa to key functional pathways, thereby elucidating the potential microbial drivers underlying functional differences.

### 4.6. QPCR Quantification of Gut Protists and Bacterial Phyla and Protist–Bacteria Correlation Analysis

The same total genomic DNA samples used for bacterial 16S rRNA gene amplicon sequencing were used as templates for qPCR quantification of selected gut protists, allowing protist abundance and bacterial ASV relative abundance to be compared using matched biological replicates. Three dominant gut protist genera, including *Cononympha*, *Holomastigotoides*, and *Pseudotrichonympha*, were selected for qPCR quantification because they represent major flagellated protist lineages previously reported in the hindgut protist community of *C. formosanus* [22].Genus-specific primers were designed based on 18S rRNA gene sequences of these protist taxa reported by this study. The *C. formosanus RPS18* gene was used as the host reference gene for qPCR normalization [43]. The primers used in this study are listed in Supplementary Table S1.

The qPCR assay was performed in a 12.5 μL reaction mixture containing 0.6 μL of total genomic DNA template,6.25 μL of 2× SYBR Green qPCR Master Mix, 0.5 μL of forward primer, 0.5 μL of reverse primer, and 4.65 μL of nuclease-free water. Amplification was carried out on a Bio-Rad CFX Opus 96 real-time PCR system (Bio-Rad, Hercules, CA, USA) under the following cycling conditions: initial denaturation at 95 °C for 10 min, followed by 39 cycles of 95 °C for 10 s, and annealing/extension at the primer-specific temperature for 15 s. After amplification, melting-curve analysis was performed from 65 °C to 95 °C in 0.5 °C increments to verify the specificity of the qPCR products. A melting-curve analysis was performed after amplification to verify the specificity of the qPCR products [54]. Each reaction was performed with four technical replicates.

Relative quantification of the selected protist genera was performed using the 2^−ΔΔCt^ method. The ΔCt value represented the difference in threshold cycle values between the protist-specific target and the reference gene (ΔCt = Ct_target protist_ − Ct_reference gene_). The ΔΔCt value was calculated by subtracting the mean ΔCt value of the worker group from the ΔCt value of each sample (ΔΔCt = ΔCt_sample_ − mean ΔCt_worker group_). The resulting 2^−ΔΔCt^ value indicated the relative abundance of each protist genus compared with the worker reference level [55]. The worker and soldier differences for the three protist genera were tested using two-sided Wilcoxon rank-sum tests, followed by BH correction.

For bacterial taxa, the relative abundances of six selected protist-associated or termite-gut bacterial genera, including *Endomicrobium*_A, *Azobacteroides*, *Treponema*_D, *Treponema*_H, *Termitinema*, and *Breznakiella*, were obtained from the unrarefied, taxonomically annotated ASV table. These bacterial groups were selected because several lineages are known to be associated with termite gut flagellates or termite hindgut symbiotic microhabitats, particularly *Azobacteroides* associated with *Pseudotrichonympha* and spirochaete lineages associated with gut protists [9,34,56]. ASV counts were first normalized to relative abundance by dividing each ASV count by the total sequencing depth of the corresponding sample. The relative abundance of each target bacterial genus was then calculated by summing all ASVs assigned to that genus.

Spearman’s rank correlation analysis was performed to evaluate associations between qPCR-derived protist abundance and bacterial relative abundance across the matched biological replicates. To explore caste-associated patterns in these protist–bacteria relationships, correlations were further calculated separately for worker and soldier samples. *p*-values from the overall correlation analysis were adjusted using the BH false discovery rate method. The 18 overall correlation tests were corrected using the BH method, with adjusted *q*-values < 0.05 considered significant.

Phylum-specific qPCR was further performed for Actinobacteriota, Bacteroidota, and Firmicutes using the same total genomic DNA samples. The qPCR procedures and relative quantification using the 2^−ΔΔCt^ method were the same as those described above for gut protists. The 16S rRNA gene sequencing-derived relative abundances of these three phyla were obtained directly from the phylum-level taxonomic composition table. For comparison between the two approaches, the qPCR-derived and sequencing-derived values were independently normalized to the mean value of the worker group. The worker and soldier differences were evaluated using two-sided Wilcoxon rank-sum tests, followed by BH correction across the six comparisons [57].

## Figures and Tables

**Table 1:** Raw data statistics after processing in gut samples of workers and soldiers of *C. formosanus*.

	Input	Filtered (%)	Denoised (%)	Merged (%)	Non-Chimeric (%)	Non-Singleton (%)
Worker_1	70,666	64,845 (91.76%)	63,613 (98.10%)	58,669 (92.23%)	51,406 (87.62%)	51,190 (99.58%)
Worker_2	69,336	62,518 (90.17%)	61,350 (98.13%)	56,191 (91.59%)	48,382 (86.10%)	48,117 (99.45%)
Worker_3	72,217	65,506 (90.71%)	63,869 (97.50%)	57,560 (90.12%)	48,924 (85.00%)	48,614 (99.37%)
Worker_4	74,467	67,174 (90.21%)	65,303 (97.21%)	57,208 (87.60%)	47,641 (83.28%)	47,179 (99.03%)
Worker_5	75,596	68,373 (90.45%)	66,924 (97.88%)	61,363 (91.69%)	53,366 (86.97%)	53,052 (99.41%)
Soldier_1	69,180	62,415 (90.22%)	61,118 (97.92%)	55,507 (90.82%)	43,520 (78.40%)	43,194 (99.25%)
Soldier_2	67,096	59,879 (89.24%)	59,084 (98.67%)	55,895 (94.60%)	49,075 (87.80%)	48,968 (99.78%)
Soldier_3	68,047	61,903 (90.97%)	60,802 (98.22%)	56,725 (93.29%)	48,616 (85.70%)	48,452 (99.66%)
Soldier_4	70,176	62,894 (89.62%)	61,550 (97.86%)	55,916 (90.85%)	43,648 (78.06%)	43,404 (99.44%)
Soldier_5	64,916	58,444 (90.03%)	57,199 (97.87%)	52,310 (91.45%)	41,809 (79.93%)	41,558 (99.40%)

**Table 2:** Summary of 16S rRNA sequencing and ASV overview of the gut of workers and soldiers of *C. formosanus*.

	ASV	Phylum	Class	Order	Family	Genus
Overall	8592	19	29	70	132	231
Workers	5360	17	25	63	115	190
Soldiers	3902	17	25	53	92	151

## Data Availability

The sequencing data were uploaded to NCBI Database under accession number PRJNA1458110 (https://www.ncbi.nlm.nih.gov/bioproject/PRJNA1458110; accessed on 13 August 2026).

## Supplementary Materials

The following supporting information can be downloaded at: https://www.mdpi.com/article/10.3390/ijms27167297/s1.
