# Supplementary material for: Caste-Associated Gut Microbial Diversity and Predicted Functional Profiles in Coptotermes formosanus
Source: Int J Mol Sci. 2026 Aug 15;27(16):7297. doi: 10.3390/ijms27167297 (PMC13513303; doi:10.3390/ijms27167297)
Supplement: Supplementary file 1 [file ijms-27-07297-s001.zip › ijms-4441367-supplementary.pdf]

Supplementary Table S1. Primers used for qPCR quantification of gut protists and bacteria

Target	Primer name	Primer sequence (5′–3′)	Amplicon length (bp)	Annealing temperature (°C)	Reference
RPS18	RPS18-F	CGATGGCAAGAGGAAGGTTAT	107	60	[54]
	RPS18-R	CCCGCTTATCCAGATCAATGT			[54]
Cononympha	Cono-F	ATTGGGTGAAGTGCGAAAGG	115	56.1	This study
	Cono-R	CCTCCCGCTCTTCTACCCAT			This study
Holomastigotoides	Holo-F	CTTGTGGCTCAATCTGACT	142	61	This study
	Holo-R	CGACGCATGACAGATCAAT			This study
Pseudotrichonympha	Pseudo-F	GGGTTAATTCTCAAGGTGGA	185	60	This study
	Pseudo-R	TATTCGCGTTGGACTGTG			This study
Actinomycetota	Actino235	CGCGGCCTATCAGCTTGTTG	166	60	[55]
	Eub518	ATTACCGCGGCTGCTGG			[55]
Bacteroidota	Cfb319	GTACTGAGACACGGACCA	181	60	[55]
	Eub518	ATTACCGCGGCTGCTGG			[55]
Bacillota	Lgc353	GCAGTAGGGAATCTTCCG	156	60	[55]
	Eub518	ATTACCGCGGCTGCTGG			[55]
